# Adrenalectomy for solitary metastasis of Hepatocellular carcinoma post liver transplantation: Case report and literature review

**DOI:** 10.12669/pjms.324.10339

**Published:** 2016

**Authors:** Imran Khan Jalbani, Syed M Nazim, Muhammad Usman Tariq, Farahat Abbas

**Affiliations:** 1 Imran Khan Jalbani, Department of Surgery, Aga Khan University, Karachi, Pakistan; 2 Syed M Nazim, Department of Surgery, Aga Khan University, Karachi, Pakistan; 3 Muhammad Usman Tariq, Department of Pathology, Aga Khan University, Karachi, Pakistan; 4 Farahat Abbas, Department of Surgery, Aga Khan University, Karachi, Pakistan

**Keywords:** Adrenalectomy, Hepatocellular carcinoma, Liver transplantation, Metastasis

## Abstract

Liver transplantation (LT) is the treatment of choice for localized hepatocellular carcinoma (HCC) associated with cirrhosis. Extra hepatic metastasis is the most common cause of death in these patients. There is very little evidence regarding the natural history and treatment options for patients developing HCC recurrence after LT. Surgical resection offers a unique opportunity for solitary metastasis. We report a 61 year old male with solitary right adrenal metastasis 15 months post LT which was managed with open adrenalectomy. The patient is alive and disease free 24 months after the surgery. The case, histo-pathological findings and literature review is discussed.

## INTRODUCTION

Hepatocellular carcinoma (HCC) is the most common tumor of liver parenchyma and the fifth most common cancer in the world.^1^ The epidemiological distribution of HCC varies across the globe being the highest in south East Asia and sub-Saharan Africa.^2^ Chronic liver disease (CLD) and HCC are the consequences of viral hepatitis and nearly 17 million in people in Pakistan are affected by viral hepatitis.^3^

Liver transplantation (LT) is treatment of choice for CLD and localized hepatocellular carcinoma.^4^ The commonest cause of death in patients with HCC is extra-hepatic metastasis which is usually multifocal.^5^ Surgical resection offers a unique opportunity for solitary metastasis after LT.^5^ To our knowledge solitary adrenal metastasis is a rare finding and the available data is limited to few case reports and there is no consensus on its management. Herein, we report a case of right adrenal metastasis of HCC fifteen month post LT, patients is alive and doing well 24 months after adrenalectomy.

## CASE REPORT

A 61-year-old gentleman with controlled diabetes mellitus and 13 years history of hepatitis C and chronic liver disease underwent live non related LT in October 2012 in China for hepatocellular carcinoma lesion measuring 3.5cm on preoperative imaging. The pre-operative serum alpha fetoprotein (AFP) values were within normal limits. Final histopathology of resected liver showed a focus of hepatocellular carcinoma grade II, with tumor size of 3 cm without any major vascular involvement. He did not receive any pre or post- transplant chemotherapy. Immunosuppressant treatment comprised of FK506 2.5mg and mycophenolate mofetil (Celcept) 750mg twice daily

**Fig.1:**
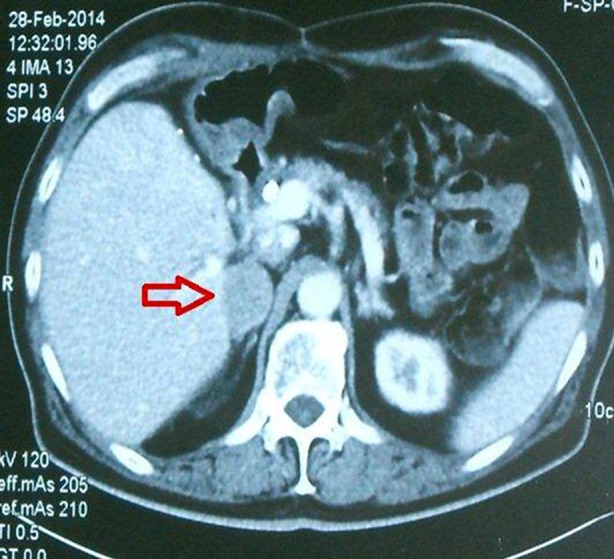
Axial images of contrast enhanced CT, showing normal transplanted liver, spleen, left kidney and right adrenal mass abutting liver (arrow).

Unfortunately, the patient did not follow a standardized surveillance protocol and was referred to us fifteen months post LT with an ultrasound scan revealing a right adrenal mass with normal serum AFP (1.0 IU/ml). He denied any history of weight loss, abdominal pain or hyper adrenergic symptoms. Contrast enhanced computerized tomography (CT) of chest and abdomen showed normal transplanted liver and an enhancing right adrenal mass with central necrosis measuring 30 x 33 x55 mm without any evidence of distant disease (Fig.1). A positron emission tomography (PET) scan confirmed the hyper metabolic lesion, raising suspicion of malignancy with standardized uptake value (SUV) of 4.5. The metabolic work-up was negative for pheochromocytoma, Cushing syndrome and Conn’s syndrome.

**Fig.2:**
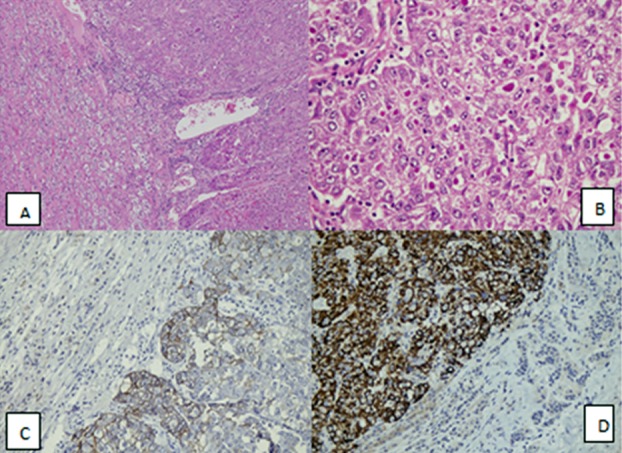
A: Tumour cells infiltrating into the normal adrenal gland parenchyma at the periphery H&E, 100 xM). B: Large lobules of neoplastic cells surrounded by rich sinusoidal network. Intracytoplasmic eosinophilic inclusions are also seen in large size, polygonal cells (H&E, 400 x M). C: Tumor cells (Right) staining positive for Cytokeratin CAM5.2 immunohistochemical stain while normal adrenal tissue (Left) are negative (200xM). D: Diffuse Hep-par1 positive immunohistochemical stain membranes and cytoplasmic (left) while normal adrenal tissue (right) is negative (H&E, 400 xM).

He underwent a right open adrenalectomy in March 2014 through right flank incision and histopathology revealed metastatic HCC with angio-lymphatic invasion (Fig.2). The patient made good recovery and was discharged. However he was advised to switch to Everolimus from FK506 as it decreases chances of HCC recurrence. After almost 39 months post-transplant he is doing well and under close surveillance.

## DISCUSSION

Liver transplantation (LT) is currently the best available option for select cases of HCC with cirrhosis and has shown to improve the overall survival.^4^ A careful follow up is needed in these patients due to possibility of recurrence not only in the graft but also in extra-hepatic organs.^1^ The recurrence of HCC after liver transplant is reported to be as high as 10%, with studies reporting most of extra hepatic recurrence within first year of transplant.^6^,^7^

There is very little evidence in literature regarding the natural history, effects of medical or surgical treatment and predictors of survival in patients who develop HCC recurrence after LT. Management of single site metastasis of HCC after LT lacks the consensus but certainly depends on various factors like, site, size of tumor, tumor resectability, patient’s functional status, comorbid medical conditions, biochemical markers (AFP) and presence or absence of HCC in the transplanted liver.^8^

In clinical practice, adrenal gland is an uncommon site of metastasis from HCC with incidence ranging from 1- 2.4%. Few autopsy reports have showed that it is the second site of hematogenous spread from HCC after lung, with reported incidence of 8.4%.^9^

The experience of surgical excision (adrenalectomy) for metastasis is limited to mainly case reports and these lesions can also be managed with other non-surgical modalities. Combined Radiofrequency ablation (RFA) with trans-arterial chemoembolization (TACE) was described in a series from Japan for adrenal metastasis from HCC and showed it to be a safe option which can lengthen survival.^9^

In an analysis of 30 patients who had adrenal metastasis from HCC, Park JS et al.^10^ reported adrenalectomy in five patients, non-surgical treatment such as TACE or chemo + radiation therapy in 19 patients. Six patients did not receive any treatment. They reported a median survival of 11 months. They also observed that the incidence of metastasis is higher on the right adrenal gland compared to left side. They concluded that adrenalectomy for metastatic HCC results in improved patient survival especially those in good general condition.

In a recently published study from Korea, Ha TY et al.^11^ studied 26 patients with metachronous adrenal metastasis in patients who underwent liver resection (19 patients) or LT (7 patients). All patients had adrenal recurrence within 18 months and underwent adrenalectomy. The mean diameter of tumor was 3.4±1.8 cm in LT group. The five year survival after adrenalectomy was 20.3%. Our case was found to have HCC of resected liver following transplantation and had metachronous adrenal metastasis 15 months post LT which was managed successfully by surgical excision.

Recurrence after LT does reduce overall survival. Several factors have been identified that contribute to increased recurrence and affect the survival. These include >1 tumor, size > 5 cm and recurrence within one year.^9^

Uka K et al.^12^ reported a cumulative survival rate of 21.7%, 14.2% and 7.1% respectively one, two and three years after the initial diagnosis of extra hepatic metastasis in patients with HCC with a median survival of 4.9 months (range, 0-37 months). Roayaie S et al. in their series have shown that patients who undergo resection or ablation of a recurrence had that.^8^

Serum AFP is a good indicator for HCC metastasis.^7^ Our case was unique in that it had normal serum AFP levels both before the LT and after adrenal metastatic despite of considerable sized tumor. The patient is presently alive and disease free. He is under regular surveillance with CT /PET scan along with tumor marker.

## CONCLUSION

We believe that surgical excision (adrenalectomy) should be considered for selected LT patients with solitary adrenal metastasis from HCC. Further evidence is needed to develop definite recommendations.
